# A Quantitative Analysis of Undisclosed Conflicts of Interest in Pharmacology Textbooks

**DOI:** 10.1371/journal.pone.0133261

**Published:** 2015-07-27

**Authors:** Brian J. Piper, Hassenet M. Telku, Drew A. Lambert

**Affiliations:** 1 Department of Basic Pharmaceutical Sciences, Husson University School of Pharmacy, Bangor, Maine, 04401, United States of America; 2 Department of Pharmacy Practice, Husson University School of Pharmacy, Bangor, Maine, 04401, United States of America; Royal College of Surgeons, IRELAND

## Abstract

**Background:**

Disclosure of potential conflicts of interest (CoI) is a standard practice for many biomedical journals but not for educational materials. The goal of this investigation was to determine whether the authors of pharmacology textbooks have undisclosed financial CoIs and to identify author characteristics associated with CoIs.

**Methods and Findings:**

The presence of potential CoIs was evaluated by submitting author names (N = 403; 36.3% female) to a patent database (Google Scholar) as well as a database that reports on the compensation ($USD) received from 15 pharmaceutical companies (ProPublica’s Dollars for Docs). All publications (N = 410) of the ten highest compensated authors from 2009 to 2013 and indexed in Pubmed were also examined for disclosure of additional companies that the authors received research support, consulted, or served on speaker’s bureaus. A total of 134 patents had been awarded (Maximum = 18/author) to textbook authors. Relative to *DiPiro’s Pharmacotherapy*: *A Pathophysiologic Approach*, contributors to *Goodman and Gilman’s Pharmacological Basis of Therapeutics* and *Katzung’s Basic and Clinical Pharmacology* were more frequently patent holders (OR = 6.45, *P *< .0005). Female authors were less likely than males to have > 1 patent (OR = 0.15, *P* < .0005). A total of $2,411,080 USD (28.3% for speaking, 27.0% for consulting, and 23.9% for research), was received by 53 authors (Range = $299 to $310,000/author). Highly compensated authors were from multiple fields including oncology, psychiatry, neurology, and urology. The maximum number of additional companies, not currently indexed in the Dollars for Docs database, for which an author had potential CoIs was 73.

**Conclusions:**

Financial CoIs are common among the authors of pharmacology and pharmacotherapy textbooks. Full transparency of potential CoIs, particularly patents, should become standard procedure for future editions of educational materials in pharmacology.

## Introduction

Conflicts of Interest (CoIs) may occur when an individual’s professional responsibilities conflict with their personal interests or when their professional responsibilities compete (e.g., clinician versus researcher). The credibility and public trust in published materials depends on how fully and transparently CoIs are handled. Many, but not all, reputable biomedical journals have developed detailed policies over the past decade requiring authors to disclose financial CoIs [1–3]. Authors with a CoI are more likely to report findings that are consistent with the interests of the research sponsor of drugs for treating cardiovascular diseases [4], cancer [5–6], and psychiatric disorders [7–8].

Recently, the author of a widely employed psychopharmacology textbook [9] listed the companies that he has consulted for, served on the speaker’s bureau, or received support for travel or research. This prompted the examination of other general pharmacology textbooks which revealed that CoIs are typically unreported [10–13]. Females who were the first or senior author of empirical reports in oncology were both more likely to declare the source of their funding and less likely to report support from industry [14]. Therefore, the objectives of this report were to: 1) determine whether the authors of widely used educational and reference materials in pharmacology have CoIs; and 2) to identify whether there are differences in the qualitative and quantitative aspects of CoIs based on the textbook or author characteristics.

## Materials and Methods

### Ethics Statement

This investigation involved evaluating potential CoIs by submitting author names to multiple databases. As such, the chairperson of the Husson IRB indicated that this did not constitute human research. Although all information reported in this study is publically available, the names of individual authors are not listed in the figures or tables. Potentially sensitive information may be obtained by contacting the authors.

### Procedures

The most recent editions of four commonly used books were selected. *Goodman and Gilman’s Pharmacological Basis of Therapeutics* [10] (PBT, 12th edition, 2011) is a pharmacology reference with a distinguished history. PBT was first published in 1941 and has historically been considered the “blue bible of pharmacology” [15]. All areas of pharmacology are represented but PBT includes a strong emphasis on medical chemistry and neuropharmacology. PBT is widely used in diverse areas of medicine as well as in the training of pharmacists and research pharmacologists. *Katzung’s Basic and Clinical Pharmacology* [13] (BCP, 12^th^ edition, 2012) is a highly readable textbook commonly utilized in the medical and allied health fields. The longest section in BCP is devoted to chemotherapeutic drugs. *DiPiro’s Pathophysiology*: *A Pathophysiologic Approach* [12] (PAPA, 9^th^ edition, 2014) is a cornerstone of the pharmacy curriculum. The greatest number of chapters in PAPA are on infectious diseases. As psychiatry has received particular attention for CoIs [16–18], the author of a collection of resources including Stahl’s Essential Psychopharmacology [9] (SEP, 4^th^ edition, 2013) was also included. Authors names from PBT, BCP, PAPA, and SEP, with the exception of those responsible for the introductory material/general principles (Section I in PBT and BCP), were entered into three databases.

#### 1. Google Scholar

Each author’s name (N = 403) and “patent” was input into the Google Scholar (http://scholar.google.com/) search engine. The checkbox “include patents” was selected to identify patents where the author was listed as an inventor or co-inventor published from 1995 until present (2014). This broad window was selected because U.S. patents granted from mid-1995 provide protection for up to seventeen years which would include the period during which the chapters were originally authored. For the present purposes, “patent” is inclusive of both applications and an issued patent as both constitute a potential CoI [1]. Names of individuals with at least one patent were submitted to a second database (http://www.freepatentsonline.com/). The primary dependent measure was the presence or absence of patents although the quantity of patents with unique titles was also recorded.

#### 2. ProPublica’s Dollars for Docs (PDD)

The database by ProPublica (http://projects.propublica.org/docdollars/) currently reports on the compensation received from from fifteen pharmaceutical companies. Most companies began contributing data in 2009 (Cephalon, Eli Lilly, GlaxoSmithKline, Merck, and Pfizer) or 2010 (Allergan, AstraZeneca, Johnson and Johnson, Novartis, Valeant and Novartis). The service provided by each author from 2009 to 2012 is listed as research, speaking, consulting, meals, travel, other, or a combination. One search was conducted in the state of the author’s employer and another nationwide in order to identify remuneration that was associated with a practice site in another state. If a range of values was provided (e.g., $90,000–100,000) than the mid-point (e.g., $95,000) was entered. The default setting is to only include values > $250. Only health care providers (i.e. MD or PharmDs) with a United States affiliation were eligible to have a PDD entry (N = 339). Dependent measures included the presence or absence of a PDD entry and the total compensation received.

#### 3. Pubmed

Additional search of the ten highest compensated authors was completed using Pubmed (http://www.ncbi.nlm.nih.gov/pubmed). CoI information was extracted from manuscripts (N = 410, Min = 7, Max = 114/author) published in the past five years (2009 to 2013) to identify any additional companies (biotechnology, medical device, or pharmaceutical) not currently covered by PDD. No adjustments in the total number of companies were made for companies that have subsequently merged, split, or are no longer solvent.

### Data-Analysis

Statistical analysis was conducted using Systat (San Jose, CA), version 13.1 (see also: S1 Dataset). An alpha < .05 was considered significant but statistics that reached more conservative thresholds (e.g., .0005) were also noted. Analyses examined whether author characteristics (the textbook contributed to, whether an author contributed to an earlier edition of the same textbook, highest professional degree, country/state of residence, sex) were associated with CoIs. In ambiguous instances (e.g., only the first initial provided), author sex was determined by consulting the National Plan and Provider Enumeration System or a general internet search. Total compensation was ranked from most to least and the top ten authors were examined separately. As authors who are also editors have the potential to exert substantial influence on textbook content, the highest compensated author/editor was also determined. Non-parametric analyses were conducted with a chi-square (e.g., presence of a PDD entry) or the Odd’s Ratio (OR). Figures were prepared with Graphpad Prism (La Jolla, CA), version 6.04. Variability was expressed as the SEM. Potential inconsistencies between CoIs identified in PDD and Pubmed were defined as instances where a company or activity was listed in a published manuscript but not in PDD for a company that supplied data to PDD.

## Results

### Author Characteristics

**Fig 1:**
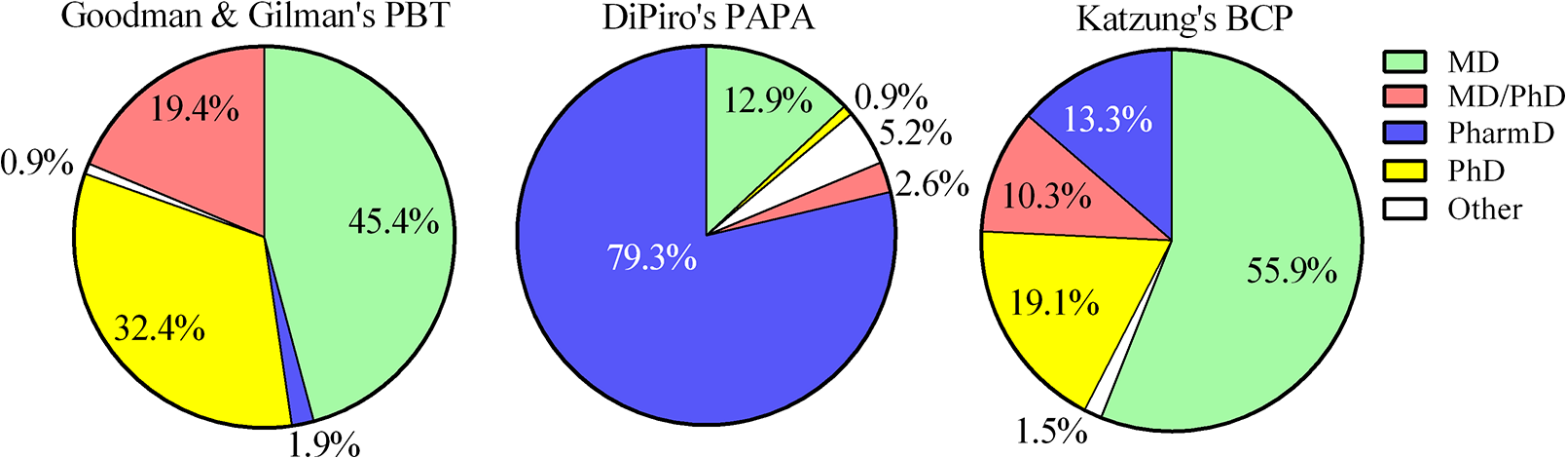
Highest academic degree of the authors by textbook.

There were some similarities as well as differences among the three multi-contributor pharmacology books (note that SEP is a single-author textbook and is included in the following sections). A greater proportion of the PAPA authors were female (52.6%) than either BCP (19.7%, χ^2^(1) = 22.42, *P* < .0001) or PBT (11.2%, χ^2^(1) = 52.38, *P* < .0001). The majority of BCP authors had an MD degree whereas over three-quarters of PAPA had a PharmD degree (Fig 1). The preponderance of authors contributed a single chapter (PAPA = 92.6%, PBT = 87.9%, BCP = 83.3%). Slightly less than half of PBT authors (44.9%) also contributed to the previous edition of this book which was significantly lower than the BCP (87.9%, χ^2^(1) = 31.84, *P* < .0001) or the PAPA (80.9%, χ^2^(1) = 44.62, *P* < .0001) authors. Less than one-tenth of authors had affiliations outside of the U.S. (PBT = 8.4%, BCP = 9.1%, PAPA = 4.8%).

### Patents

**Fig 2:**
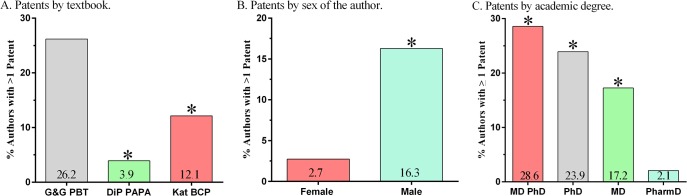
Patents by author characteristics (* *P* < ·05). Goodman and Gilman’s Pharmacological Basis of Therapeutics (G&G PBT), DiPiro’s Pharmacotherapy: A Pathophysiological Approach: (DiP PAPA), Katzung’s Basic and Clinical Pharmacology (Kat BCP).

The authors had been granted 134 patents (Maximum/author = 18). Examples of patent titles that showed a particular overlap with the content of a chapter are shown in Table 1. The percentage of authors having at least one patent (11.4%) differed based on the book. BCP was larger than PAPA (χ^2^(1) = 6.38, *P* < .025). PBT was also greater than PAPA (χ^2^(1) = 37.01, *P* < .0001) and BCP (χ^2^(1) = 4.89, *P* < .05, Fig 2A). A complete listing of the patent titles for each textbook may be obtained from B.J.P. Females were less likely than males to have at least one patent (OR = 0.15, *P* < .0005, Fig 2B). Highest academic degree also was associated with having > 1 patent with PharmDs being significantly (*P* < .0001) less likely other degree holders (Fig 2C).

**Table 1:** Example chapter and patent titles among authors of DiPiro’s Pharmacotherapy: A Pathophysiological Approach, Goodman & Gilman’s The Pharmacological Basis of Therapeutics, or Katzung’s Basic and Clinical Pharmacology.

Title of Chapter	Title of Patent(s)
Asthma	Genetic Predictor of Efficacy of Anti-Asthmatic Agents for Improving Pulmonary Function
Parkinson’s Disease	Composition and Method for Decreasing Neurologic Symptomatology
Opioids, Analgesia, and Pain	Methods for Treating Pain; Peripherally Active Anti-Hyperalgesic Opiates; Method of Producing Analgesia
Pharmacotherapy of Diabetes	Insulin-Dependent Diabetes Mellitus-Specific Chimeric Polypeptides
Helminth Infections	Hookworm Anticoagulant
Antiviral Agents	Predicting Probabilities of Achieving a Desired Minimum Trough Level for an Anti-Infective Agent
Dermatological Pharmacology	Topical Preparations & Therapy for Head Lice
Adrenoceptor Antagonist Drugs	Selective Antagonists of A_2B_ Adenosine Receptors
Nitric Oxide	Protein Inhibitor of Neuronal Nitric Oxide Synthase
Local Anesthetics	Guanidine Compounds as Anesthetics and for Treatment of Nervous System Disorders

### Compensation

PBT (30.4%) were more likely than PAPA authors to have a PDD entry (OR = 4.84, *P* < .0005). Similarly, BCP (26.0%) authors also had more entries than PAPA (OR = 3.88, *P* < .0005). A PDD entry was less common among female (4.7%) than male (22.6%, OR = 0.17, *P* < .0005) authors. PharmDs (4.9%) were less likely than either MDs (32.1%, χ^2^(1) = 39.62, *P* ≤ .0001) or MD/PhD (29.0%, χ^2^(1) = 19·40, *P* ≤ .0001) authors to have a PDD entry.

A total of $2,411,080 was received by 53 authors (6 females and 47 males, Range = $299 to $310K/author). The largest category of support was for speaking (28.3% of total compensation/author) followed by consulting (27.0%), research (23.9%), travel (8.4%), combination (6.2%), and other (2.4%).

**Fig 3:**
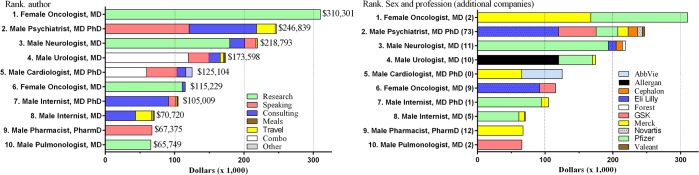
Compensation received from the top ten authors by funded activity (left) and specific company (right).

Over half (62.1%) of the total remuneration was to the top ten highest compensated authors. These authors and the activities they received compensation for are shown in Fig 3A. The most compensated authors represented a variety of specialties including oncology, psychiatry, neurology, urology, and cardiology. Four authors received the vast majority (>80%) of their support for research although two others were compensated primarily for consulting and another exclusively for speaking. Fig 3B shows the companies providing compensation. Three authors received the majority of their support from Merck and three more from Pfizer. Four PBT and three BCP authors were represented among the top ten. The highest ranked author/editor was at position #29 with $17,244 in compensation.

Examination of recent manuscripts revealed many additional companies (Mean = 12.6 ± 6.9; Median = 7.5, Min = 0, Max = 73) that provided compensation to the authors. Inconsistencies between PDD and the publications indexed in Pubmed were infrequent (10/410 manuscripts or 2.4%).

Additional analyses were conducted among the subset of authors with a PDD entry. The average amount received was $45,492 ± 8,846 (Median = 24,470). This value was not significantly different based on the textbook (PBT = $56,888 ± 18,050; BCP = $33,415 ± 8,655; PAPA = $31,038 ± 9,783) or author gender (Males = $41,228 ± 7,901, Females = $78,894 ± 49,380). However, MDs ($47,724 ± 11,477) received more money than PharmDs ($18,162 ± 7,786, *t*(37.6) = 2.13, *P* < .05). Dual-degree (MD/PhD) holders ($65,526 ± 27,108) did not receive significantly more than others.

## Discussion

The primary goal of this investigation was to determine if pharmacology authors had appreciable CoIs or what could be reasonably perceived as financial CoIs. Based on detailed examination of three complementary resources, patents, PDD, and the CoI section of published manuscripts, the answer is clearly in the affirmative. PBT, BCP, and PAPA currently do not consistently report financial CoIs. In contrast, the preface of SEP notes the many companies that have provided remuneration. These resources consistently neglect to disclose patents. Overall, the financial CoIs were not insubstantial for the subset of textbook authors that had either a patent or a PDD entry. For example, two authors of a chapter in the inflammation section of PBT [10] together received 23 patents, many of which were highly relevant to the subject matter addressed. Similarly, the maximum compensation/author from pharmaceutical companies was over $310,000 which was distributed over two years. Notably, the compensation received per author does appear to be lower than that reported for a prominent “ethically challenged” [18] psychiatrist. Further, the frequency of ties with pharmaceutical companies in pharmacology textbooks appears less widespread than in other influential resources like working group members responsible for recent editions of the Diagnostic and Statistical Manual of Mental Disorders [16]. Although this may be reason for some optimism, at least other resources like the Diagnostic and Statistical Manual are now cognizant of CoI. Given the large evidence base that the funding source has an appreciable impact on what biomedical information is presented and how favorably it is portrayed [5–8,19,20], the systematic omission of financial CoI in three of the four textbooks evaluated is simply an unacceptable practice.

The second objective of this report was to determine the factors associated with CoIs. Both patents and the compensation frequency were more common in pharmacology (PBT & BCP) relative to pharmacotherapy (PAPA) textbooks. Pharmacists were less likely than physicians or PhD trained pharmacologists to have financial CoIs in the form of a patent. Similarly, PharmDs were less likely than other professional degree holders to have received compensation from pharmaceutical companies according to PDD. This finding is congruent with a prior analysis from the PDD [21]. Possibly, individuals with aspirations of becoming inventors or conducting pharmacology research may choose to receive their training in medicine or as scientists rather than other allied health fields. Alternatively, as the preponderance of authors were affiliated with institutions of higher learning, medical schools may have greater intellectual property infrastructure than pharmacy schools to support the patent application process. Although not the primary objective of this report, the finding that males outnumbered females 4:1 in BCP and 8:1 in PBT as authors is not just noteworthy, but also concerning. This is likely reflective of the persistent inequality in publishing in academic medicine [22]. There were also pronounced sex differences in the likelihood of being a patent holder. Females accounted for only three of the top thirty highest compensated authors (#26, #6, and #1).

There are some reasons to believe that the total remuneration reported in Fig 1A is an under-estimate of the compensation provided by companies to authors. Not all companies began reporting to PDD in the same year or report all categories of support [21,23]. As many as 73 additional companies/author were identified by examining the acknowledgments section, the CoI section, or the International Committee of Medical Journal Editors (ICMJE) CoI form [1] and these are not currently included among the fifteen with detailed monetary values and supported activities in PDD. We are cautiously optimistic that future projects of this type will benefit from laws like the Physician Payments Sunshine Act which will mandate more widespread disclosures and with a very low threshold [24,25]. Importantly, even quantifying the number of companies an author is associated with is impeded by innocuous sounding foundation names that are listed in the acknowledgments section (e.g., the Foundation for Lung Cancer: Early Detection, Prevention, and Treatment) which are largely fronts for commercial entities [26]. While self-reported disclosure will continue to be an integral element of any comprehensive of CoI policy, the authors who received a modest honorarium for contributing a textbook chapter but failed to list this on the ICMJE form [1] did not go unnoticed. This omission is reflective of either selective recall or more wide-spread under-reporting. Although the majority of authors were eligible to have a PDD entry, PDD does not include authors located outside of the U.S. or PhD scientists. Unfortunately, even the Sunshine Act currently overlooks non-physician investigators [25]. It is also noteworthy that the manuscripts of four of the top ten highest compensated authors provide information from companies that are indexed by PDD but were not identified with a PDD search. The origin of these discrepancies is currently unclear but we can only hope that these simply reflect authors using a longer window of disclosure than the PDD currently employs.

In the event the editors of these and other medical educational resources provide a detailed account of all relevant potential financial and non-financial CoIs in future editions, one then may speculate what impact this will have. Although there is not an extensive body of empirical evidence [27,28], we currently believe that disclosure is a good general practice which may have more of an influence on the experienced course director selecting materials for their classes than on the beginning medical or pharmacy student. Furthermore, requiring authors to provide COI statements will raise awareness of COI issues with undergraduate students and encourage them to make their own disclosures later in their professional career and to look for COIs when they read prescribing advice. Similarly, greater transparency in disclosing patents might encourage some editors to make different choices when selecting authors to contribute chapters.

Three limitations and future directions of this report should be clearly noted. First, although the resources selected for this study are highly influential, they originate from a single country. Further study with other pharmacotherapy textbooks [11] or with pharmacology books with authors outside of the United States [29] would be beneficial, particularly if, or when, resources like PDD become more widespread internationally. Second, the presence and number of patents for each author was determined. Although many, perhaps the majority, are highly related to the chapter content, no effort was made to formally quantify the degree of overlap and this will require further research. Third, while this study identified many potential CoIs, we can not infer from these findings that these outside interests, either among individual authors or collectively, impacted the presentation of material in any way. Follow-up studies would be needed to untangle the contribution that financial or non-financial CoIs have on textbook content.

In conclusion, an appreciable portion of the content in pharmacology textbooks is open to the influence of undisclosed potential financial conflicts of interest. These resources are frequently consulted by many practicing physicians as well as other allied health professionals and are also integral to their education. If all authors of future editions of textbooks in pharmacology, as well as other biomedical fields, completed the ICMJE form [1] for CoI disclosures and this information were made publically available in a searchable database, this would be an appropriate first step to begin to remedy this unfortunate oversight.

## Supporting Information

S1 Dataset (XLSX)
